# Assessment of Paracetamol Usage Practices and Perceptions among Caregivers for Children Post-COVID-19 in Saudi Arabia: A Cross-Sectional Analysis

**DOI:** 10.3390/healthcare12101047

**Published:** 2024-05-20

**Authors:** Saad S. Alqahtani, David Banji, Otilia J. F. Banji

**Affiliations:** 1 Department of Clinical Pharmacy, College of Pharmacy, King Khalid University, Abha 61421, Saudi Arabia; ss.alqahtani@kku.edu.sa; 2 Department of Pharmacology & Toxicology, College of Pharmacy, Jazan University, Jazan 45142, Saudi Arabia; 3 Department of Clinical Pharmacy, College of Pharmacy, Jazan University, Jazan 45142, Saudi Arabia

**Keywords:** fever, paracetamol, caregivers, self-care, over-use

## Abstract

Caregivers often use paracetamol in the management of pediatric illnesses without a clear rationale. This study evaluated the perception and practices of caregivers regarding the use of paracetamol in children in Southwestern Saudi Arabia. This study involved a cross-sectional design involving 373 caregivers of children under twelve. The questionnaire elucidated the reasons, usage frequency, and safe usage practices. Data were analyzed using SPSS, applying Chi-square tests and logistic regression. Most caregivers were in the age range of 31–40 years, and with intermediate education. Paracetamol was mainly used for fever, generalized pain and, diarrhea, with fewer opting for other self-care measures. Caregivers with primary or less education were more likely to perceive paracetamol as safe (AOR = 2.98 (1.3–6.73), *p* = 0.009) and less inclined to check warning labels (AOR: 0.11 (0.05–0.25), *p* < 0.001) and expiry dates (AOR: 0.063 (0.027–0.14), *p* < 0.001). The caregiver’s education level significantly influenced the determination of treatment duration (χ^2^ = 21.58 (4), *p* < 0.001), dosage (χ^2^ = 30.70 (4), *p* < 0.001), and frequency of administration (χ^2^ = 17.77 (4), *p* = 0.001). In conclusion, inadequate health literacy can result in a lack of attention towards crucial safety information about pediatric paracetamol use. Hence, counselling initiatives should be undertaken to ensure the safe and effective use of paracetamol in children.

## 1. Introduction

A developing immune system, high respiration rates, and an energetic physical state make children more vulnerable to diseases such as fever, pain, and rhinitis. Elevated body temperature is triggered by a complex interplay of internal factors related to inflammation, infection, or malignancies and external factors like pathogens and toxins [1,2]. Fever, discomfort, and uneasiness usually cripple activity in children. Generally, as the first option, parents administer antipyretics and analgesics before seeking medical intervention to lessen the harmful consequences of fever [3]. A study conducted in Saudi Arabia has revealed that 84.5% of parents administer paracetamol to manage fever in their children [4]. An observational study by Kamel et al. in Jeddah, Saudi Arabia, evaluated parental knowledge and practice using ibuprofen and paracetamol. This study highlighted a lack of parental awareness of the correct dose and frequency of administration of paracetamol [5]. Furthermore, another study indicated that 93.3% of parents prefer to self-medicate their child with paracetamol to avoid spending time in pediatric clinics [6]. In addition, the recent COVID-19 pandemic has increased parental concerns and anxiety when the child develops a fever. This heightened concern is often driven by the thought that the fever could indicate a COVID-19 infection [7]. A post-pandemic study has revealed that parents are more inclined to attribute fever to COVID-19 than other symptoms like cough [8]. As a result, there has been a marked increase in the utilization of paracetamol due to fear [9]. Additionally, numerous guidelines and recommendations were put forth during the pandemic regarding antipyretic medications [10,11,12], bolstering confidence in their utilization. Furthermore, the reliance on paracetamol could be higher, as it is an easily accessible over-the-counter medication [13]. Thus, treating illnesses in children at home is often performed with the best intentions, but it can sometimes have adverse consequences.

Paracetamol, an analgesic and antipyretic, inhibits the formation of prostaglandins. The analgesic effect is also attributed to N-acylphenolamine, a paracetamol metabolite that activates the cannabinoid 1 and transient receptor potential vanilloid 1 [14].

Paracetamol is proven to be safe if administered in recommended doses. However, irrational use of paracetamol has been linked to adverse outcomes in various age groups and geographic regions [15,16]. Moreover, higher doses of paracetamol can cause hepatic and renal failure due to the formation of a toxic metabolite, N-acetyl-p-benzoquinoneimine, which depletes glutathione reserves, causing damage to hepatic and renal macromolecules [17,18]. Research conducted with caregivers at the Faisal Specialist Hospital and Research Centre in Saudi Arabia has revealed that 30% of the parents administer paracetamol in supratherapeutic doses to their sick children [16]. Intentional or accidental overdosing cannot be ruled out if the doses are not spaced adequately, adult formulations are used, and two or more medications containing paracetamol are given together, potentiating the risk of overdosage. Paracetamol-related poisoning is reported worldwide, with emergency departments in European countries such as Spain and Norway recording poisoning ranging from 4.5% to 12% [19,20]. In Saudi Arabia, almost 30% of cases of paracetamol poisoning have been reported [21], while 40–44% of cases have been noted in the United Kingdom [22]. The poison control center in the United States of America received 80,000 cases related to the use of paracetamol in 2021 alone [23]. A single supratherapeutic dose of paracetamol could cause acute poisoning, and repeated supratherapeutic doses can cause chronic poisoning. A 150 mg/kg or 100 mg/kg dose with other precipitating factors may cause acute poisoning in children [24].

We perceive that there could be potential knowledge and practice gaps which could affect the proper dosing, duration, and frequency of administration of paracetamol. Additionally, variations in parental knowledge, exposure, approach, and techniques can alter how an afflicted child is handled, potentially increasing the risk of toxicity. Moreover, due to the widespread availability and likely increased use of paracetamol post-pandemic, it is crucial to assess caregiver behaviors at periodic intervals. Therefore, this study aimed to assess perceptions and practices of caregivers in Saudi Arabia regarding the use of paracetamol for childhood illnesses.

## 2. Methods

### 2.1. Study Design

A study with a cross-sectional design was carried out, focusing on parents or guardians, collectively referred to as caregivers.

### 2.2. Ethics Approval

This study was approved by the Institutional Review Board, College of Pharmacy, bearing the number Rec-4/10/656 dated 19 May 2023.

### 2.3. Study Setting

Caregivers visiting primary healthcare centers and the outpatient pediatric department of tertiary care hospitals in the Jazan region, a part of Southwestern Saudi Arabia, were requested to participate in the study. The Jazan region has 170 primary healthcare centers (PHCs) and tertiary care hospitals. For a population of 10,000, the rate of PHCs is 1.08 [25]. PHCs were randomly selected from the compiled list using the table of random numbers.

### 2.4. Participants

We included caregivers seeking medical intervention for the child aged between 1 and 12 years willing to participate and assured them of the confidentiality of information. The inclusion criteria set was paracetamol administration by the caregiver before seeking medical attention. We excluded caregivers with a child under one year if paracetamol was not administered to their child or the child was treated with paracetamol for only one day. Data from caregivers were gathered using a convenience sampling approach between May 2023 and December 2023.

### 2.5. Sample Size

The estimation of the sample size required for assessing the prevalence of paracetamol usage as a remedy for children under twelve in the Jazan region, with a population of 1.56 million, was conducted using a statistical approach to ensure accuracy and reliability of the findings. Given the assumption that 50% of caregivers administer paracetamol to children under twelve, this study aimed for a 95% confidence level, with a margin of error set at 5% to delineate the acceptable variability in results. Utilizing the Raosoft sample size calculator, it was determined that a sample size of 385 caregivers was necessary to meet these parameters. To compensate for potential non-response, the sample size was adjusted to 405. This size is sufficient to accurately reflect the usage patterns of paracetamol within the specified confidence level and margin of error, thus providing a reliable basis for understanding the prevalence of its use in the target population.

### 2.6. Data Collection Tool

Following a thorough examination of the existing literature on the administration of paracetamol to children [26,27], a questionnaire was developed in English and scrutinized by two practicing pharmacists. The survey questionnaire was translated into Arabic to make it suitable for use among the caregivers. The questionnaire underwent a forward and back translation process between Arabic and English, facilitated by a linguist. The original questionnaire and its translated version were carefully examined to identify discrepancies. Next, an informal evaluation was conducted involving 20 caregivers who visited the same hospital in the early days of May. This review aimed to ensure that the questions were understandable, pertinent, and suitable based on the objectives of this survey. Additionally, individuals responsible for administering the questionnaire received specialized training to reduce interpersonal variations.

The survey questionnaire covered a range of essential aspects, including demographic information about caregivers (such as age, gender, location, and education), the child’s age, and inquiries about caregivers’ practices related to administering paracetamol, including questions about dosage, frequency, and duration of use. It also delved into topics concerning the safe usage of paracetamol. Additionally, the questionnaire explored the reasons caregivers brought the child to the hospital and identified the sources of information they relied on for administering paracetamol.

### 2.7. Data Processing and Analysis

The data were analyzed using SPSS Statistics for Windows, IBM, Version 23.0 (Armonk, NY, USA). Frequency measures are denoted in absolute terms along with the percentages. The relationship between the variables was analyzed using either the Chi-square test or Fisher’s exact test, depending on which was more suitable for the data. Multivariate logistic regression was performed on selected dependent variables with dichotomous options after adjusting for age and caregiver type. The level of statistical significance was set at *p* < 0.05.

## 3. Results

### 3.1. Demographic Information

**Figure 1:**
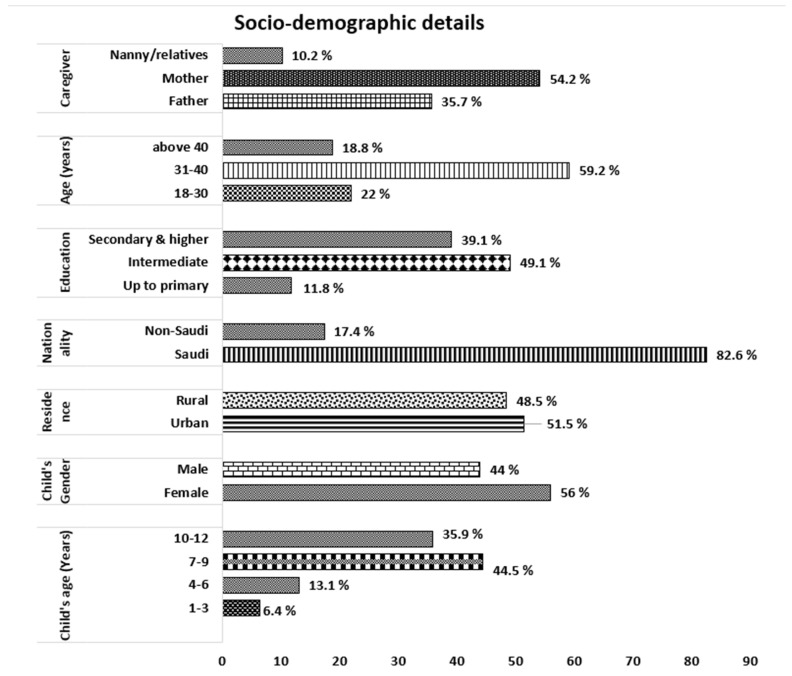
Demographic characteristics of the caregivers and the children treated with paracetamol.

Three hundred and seventy-three caregivers responded to our survey, resulting in a response rate of 88.38%. Most respondents were parents, with only about 10% of relatives or nannies. The caregivers predominantly ranged in age from 31 to 40 years, with 39.1% having secondary or higher education. Caregivers were equally split between urban and rural residents, and most were Saudis. Children aged 7–9 years and 10–12 years were the most represented age groups of the respondents (Figure 1).

### 3.2. General Perception of Caregivers about Paracetamol

Although most caregivers did not administer paracetamol in conjunction with other medications, a small number did. Of those, only 9.9% checked the contents on the label. Almost one-half of the caregivers admitted that the use of paracetamol increased post-pandemic, and an equal proportion were uncertain about whether paracetamol could be used for all pediatric health issues. Most caregivers predominately used home-stored medicines, while a few opted for tepid sponging and hydration. Paracetamol was predominately used to manage fever (50.1%), followed by headaches, vomiting, and fever associated with COVID-19 vaccination; however, its use to manage cough was comparatively low. Over one-half of the caregivers sourced paracetamol from their home supplies, with the remainder obtaining it from physicians and pharmacies. Caregivers ascertained the dose based on their personal experience and sought advice from relatives and consulting physicians. However, reading the label was a less favored option. Almost one-third of the caregivers did not receive formal counselling on the use of paracetamol (Table 1).

### 3.3. Caregiver Perceptions on the Safe Use of Paracetamol

A minority of the caregivers affirmed that paracetamol is safe. Educational level was significantly associated with the assertion that paracetamol is safe (χ^2^ = 8.44 (3), *p* = 0.037). Further, the logistic regression model demonstrated that caregivers with education up to the primary level were 2.98 times more likely to believe that paracetamol is safe (AOR = 2.98 (CI:1.3–6.73), *p* = 0.009) compared to those with secondary and higher education (Table 2).

### 3.4. Caregiver Practices Regarding the Use of Paracetamol

Most caregivers (60.6%) administered paracetamol for three days, while 19% extended administration beyond five days. Over one-third of caregivers aged between 31 and 40 years administered paracetamol for over five days. A significant association was found between educational level and the decision regarding the duration of treatment (χ^2^ = 21.58 (4), *p* < 0.001). Over a 24 h period, 64.1% of caregivers administered paracetamol every 3 h, 16.3% every 5 h, and 19.6% every 6 h. Frequency of administration over 24 h was significantly associated with education (χ^2^ = 17.77 (4), *p* = 0.001) but not with age and caretaker type. Approximately half of the caregivers gave paracetamol based on the severity of the fever, and this was more prevalent among caregivers aged between 31 and 40 years, but the association was not statistically significant. A statistically significant association was found between caregivers’ educational level (χ^2^ = 30.70 (4), *p* < 0.001) and selecting paracetamol dose based on age, body weight, and severity of fever (Table 3).

### 3.5. Practices in Reviewing Critical Safety Information

Most caregivers referred to the directions on the label, while approximately half noticed the warning. A significant association was found between caregiver type and their attention to label warnings (χ^2^ = 7.51 (2), *p* < 0.023). Among caregivers aged 31 to 40, 48.3% checked the directions on the label and 28.7% overlooked it; however, no significant relationship between age and safe practices was observed. About 32% of the caregivers with secondary or higher education followed the label directions, and a statistically significant association was found between education and checking directions on the label (Fisher exact = 15.98, *p* < 0.001) and warnings on the label (χ^2^ = 61.60 (2), *p* < 0.001). The logistic regression model revealed that caregivers with only primary education or less (adjusted odds ratio (AOR) (95% CI): 0.11 (0.05–0.25), *p* < 0.001) and those with intermediate education (AOR (95% CI): 0.19 (0.11–0.31), *p* < 0.001) were significantly less likely to check warnings compared with those with secondary and higher education (Table 4).

### 3.6. Caregivers’ Practice of Checking the Expiry Date

Approximately 73% of the caregivers with intermediate to higher education and 46.1% aged between 31 and 40 years checked the expiration date. A statistically significant association was found between education and checking the expiry date (χ^2^ = 81.34 (2), *p* < 0.001). Caregivers with primary education or less were significantly less likely to check expiry date (AOR: 0.063 (0.027–0.14), *p* < 0.001) compared to those with secondary and higher education (Table 5).

### 3.7. Reasons for Seeking Assistance

**Figure 2:**
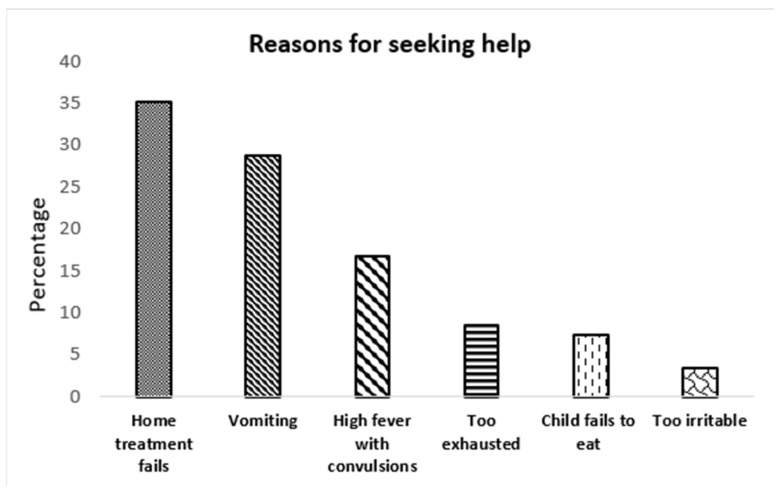
Reasons stated by caregivers for seeking medical help.

Figure 2 outlines the reasons caregivers sought help, with failed home treatment (35.2%) and vomiting (28.7%) being the most common, while a few sought assistance when the child changed their eating habits and was weak.

## 4. Discussion

This study identified some misconceptions among caregivers initiating treatment with paracetamol.

Nearly sixty percent of the caregivers in our study were in the age group between 31 and 40 years, and 22% fell within the age range of 18 and 30 years. These findings align with those of Obu et al., who reported that 90.3% of caregivers were aged between 18 and 57 years [28].

The administration of paracetamol in pediatric populations is a ubiquitous practice, with caregivers frequently utilizing it for fever management [29]. While paracetamol is considered to be safe when used in recommended doses [27,30], tolerability changes as the dose increases [27], thus inundating the body’s capacity to process it safely. In our setting, caregivers cited various reasons for administering paracetamol in children, with around half acknowledging its use for fever reduction, and this finding is consistent with previous studies. For instance, a study by De Bont et al. in the Netherlands found that paracetamol use was the highest in children aged 5 to 12 years, and the use was also seen during follow-up [31]. Similarly, a study conducted in Rome has reflected that their caregivers have given 72% of children paracetamol for fever management [32]. Furthermore, a study performed in a Northern Nigerian population revealed that 68.3% of the caregivers administered paracetamol to children for fever reduction [33]. This percentage is higher than that reported in our study, highlighting regional disparities in caregiver practices and paracetamol usage patterns.

Moreover, the use of paracetamol has increased post-pandemic, possibly due to fear and anxiety. Mattiuzzi and Lipp, in an infodemiological analysis, revealed that the use of paracetamol substantially increased compared with the pre-COVID period [34].

When respondents were asked if “paracetamol can be used for all health issues in children”, we expected the majority to respond in the negative; however, nearly one-half were unsure, and 23.1% agreed that it could be used. In line with our findings, an earlier study in Malaysia has also reported that paracetamol has been used for unintended reasons [35]. In contrast to what we observed, another study conducted in Jeddah, Saudi Arabia, reported that a little less than three-fourths of the caregivers felt that paracetamol should not be given [5]. The difference in perception could be attributed to the limited health literacy or inadequate counselling by healthcare professionals. Unfortunately, it was also observed that some caregivers had used paracetamol for vomiting and diarrhea and to manage fever/irritability associated with COVID-19 vaccination.

Approximately two-thirds of the caregivers stated that paracetamol is a safe medication; among whom 34.3% are mothers. The results of our study correspond to the findings of a Malaysian study, where 69.1% of consumers held similar views [26]. Moreover, we also found some caregivers administering other medications concurrently with paracetamol. This is not an isolated case, as a study among caregivers in Australia also reported that 45.8% gave dual therapy to their children [36]. Furthermore, caregivers with primary education and, to an extent, some with intermediate education were more likely to consider paracetamol as safe. This raises concerns about potential risks associated with this misunderstanding.

The decision about determining the dose, duration, and frequency of administration of paracetamol yielded favorable as well as concerning results. The correct dose of paracetamol should be based on body weight and not on the age or severity of the condition [37]. Unfortunately, over two-fifths of the parents determined the dose based on the severity of the fever. The study by Kamel et al. in Jeddah, Saudi Arabia, reported a similar trend, with caregivers administering paracetamol based on the severity of the condition; however, the proportion of use was lower than in our respondents [5]. In our survey, more than half of the parents administered paracetamol at inappropriate intervals, specifically every three hours, and a minority extended treatment beyond five days. In contrast, a study conducted in Palestine showed that 61.6% of caregivers administered paracetamol over 4–6 h, but 21.3% administered it every two hours [27]. The deviation in dosing decisions might be attributed to caregivers considering symptom severity a crucial factor. Hence, while comparing our results to the existing literature, we found that irregular dosing frequency and duration are widespread and not confined to a specific region. Also, the educational background of caregivers could impact informed choices regarding the appropriate use of paracetamol.

A little less than one-half of the caregivers had used their personal experience to decide the dose and frequency of administration. An Australian study revealed a similar trend despite respondents having high health literacy [38]. Interestingly, nearly thirty percent of the respondents in our study sought advice from family/friends, a response rate higher than that reported in a previous study in Saudi Arabia, where only 12.6% consulted family and 1.8% consulted friends [39]. The difference in information-seeking behavior could plausibly be attributed to the level of education in their sample population, which had 58.4% graduates, as opposed to 39% with secondary education and above in our sample.

When caregivers were asked about self-care measures for sick children, most opted for home-stored medications, with alternative methods such as tepid sponging and fluid replenishment utilized less. However, the latter helps to reduce the risk of dehydration. The preference for paracetamol among caregivers can be attributed to its over-the-counter availability, frequent physician recommendations, and caregiver familiarity with the medication.

The level of education among the caregivers significantly influences their tendency to check label warnings. Caregivers with elementary education and less and intermediate education were significant predictors, as they were less likely to check the warning on the label relative to caregivers with secondary and higher education. This coincides with the results reported in a study in Malaysia, indicating that caregivers with lower education did not refer to label information [35]. In addition, we found caregiver type significantly associated with their ability to check the warning on the label. Further, caregivers with education up to the primary level were much less likely to check the expiry date than caregivers with higher education. A study conducted on university students in Pakistan revealed that a mere 16.6% of them read the information provided on the label, thus suggesting that even among the educated, there exists a gap in reading medication labels [40]. Expired medications lose their potency and degrade into harmful chemicals, thus could cause an unintentional risk in children.

We identified caregivers seeking professional medical help only when home treatment failed. The response resonated with another study, where the use of unprescribed medicines caused a delay in seeking care [41]. On the other hand, some caregivers sought medical care when their child was frail and anorectic, suggesting a compromised nutritional status. Administration of paracetamol to a nutritionally compromised child can increase the risk of liver toxicity, as poor nutrition can diminish antioxidants such as glutathione [42,43,44].

The widespread misuse of paracetamol among caregivers, often based on the severity of fever rather than proper dosing guidelines, can lead to under or overdosing. Underdosing can prolong the illness, while more frequent and higher doses can result in hepatic complications. The misconception that “paracetamol is fit for all” may delay necessary professional medical consultation, worsening the health condition of the child. This misuse of over-the-counter medications like paracetamol can become a considerable public health challenge. Furthermore, the associated rise in emergency department visits and hospitalization can burden healthcare systems substantially. Healthcare professionals can provide medication counselling to mitigate these risks, especially for caregivers with limited health literacy. Secondly, stricter regulations on the sale and advertising of over-the-counter medications like paracetamol could help in preventing stockpiling and misuse. Such measures would collectively improve patient safety and reduce the load on an already burdened healthcare system.

This study has some limitations. Firstly, as it was a self-report, there could be a possibility of under-reporting the over-use of paracetamol, which could skew the results. Further, the number of caregivers with lower education was not proportional to the rest of the participants, which could impact generalizability. Despite these limitations, this study successfully identified several misconceptions about the use of paracetamol in children, which can help in designing educational interventions.

## 5. Conclusions

In conclusion, the observed increase in paracetamol use post-COVID-19 signifies a shift in self-care practices among caregivers. Misconceptions among caregivers led them to use paracetamol for various health issues in children. This study highlights that many caregivers perceive paracetamol as inherently safe and base their dosing decisions on the severity of symptoms rather than body weight. Relying on personal experience rather than professional guidance has led to inappropriate dosing frequencies and durations, which can adversely impact pediatric health. Moreover, caregivers with only primary or intermediate education were less likely to consult or correctly interpret medication labels. These findings reflect prevalent misconceptions about paracetamol among caregivers, necessitating educational and counselling initiatives to promote the rational and safe use of paracetamol in children.

## Figures and Tables

**Table 1:** Perceptions of caregivers on the use of paracetamol in children.

Variable	Response N (%)	Variable	Response N (%)
Can paracetamol be used for all health issues in children		Reasons for giving paracetamol	
Yes	86 (23.1)	Fever	187 (50.1)
No	102 (27.3)	Generalized body pain/earache/headache	61 (16.4)
Not sure	184 (49.3)	Diarrhea/vomiting	71 (19)
Has your use of paracetamol increased post-pandemic		Irritability due to COVID-19 vaccination	36 (9.7)
Yes	187 (50.1)	Cough	18 (4.8)
No	185 (49.6)	Source of paracetamol	
Given other medication along with paracetamol		Home-stored	195 (52.3)
Yes	59 (15.8)	Pharmacy	64 (17.2)
No	314 (84.2)	Doctor	114 (30.6)
If yes, did you check the contents		Determination of dose	
Yes	37 (9.9)	Personal experience	164 (44)
No	22 (5.9)	Relatives/friends	126 (29.9)
Type of selfcare given to your sick child		Physician	97 (26)
Home-stored medicines	267 (71.6)	Suggestions from healthcare professionals	62 (16.6)
Plenty of fluids	56 (15)	Directions on label	50 (13.4)
Tepid sponging	50 (13.4)	Received counselling	
Suggestions from healthcare professionals	62 (16.6)	Yes	253 (68)
		No	119 (32)

**Table 2:** Assessment of the association between caregivers’ perception that paracetamol is safe against demographic variables.

Independent Variable	Dependent Variable		χ^2^ (df)	*p*-Value	AOR & 95% CI	*p*-Value
	Paracetamol Is Safe					
	Yes (%)	No (%)				
**Caregiver**			0.417 (2)	0.813		
Mother	128 (34.3)	74 (19.8)			1	
Father	76 (20.4)	51 (13.7)			0.66 (0.30–1.42)	0.28
Nanny/relatives	27 (7.2)	17 (4.6)			0.89 (0.42–1.88)	0.77
**Age (Years)**			1.72 (2)	0.72		
18–30	53 (14.2)	29 (7.8)			0.94 (0.47–1.88)	0.86
31–40	131 (35.1)	90 (24.1)			0.74 (0.41–1.32)	0.31
Above 40	47 (12.6)	23 (6.2)			1	
**Education**			8.44 (3)	0.037 *		
Up to primary education	20 (5.4)	7 (1.9)			2.98 (1.3–6.73)	0.009 *
Intermediate	104 (27.9)	68 (18.2)			1.20 (0.76–1.88)	0.42
Secondary and higher	61 (16.4)	51 (13.7)			1	
**Domicile**			0.20 (1)	0.65		
Urban	121 (32.4)	71 (19)			1	
Rural	110 (29.5)	71 (19)			1.18 (0.76–1.82)	0.44
**Nationality**			0.87 (1)	0.34		
Saudi	140 (37.5)	79 (21.2)			1	
Non-Saudi	91 (24.4)	63 (16.9)			1.09 (0.71–1.67)	0.67

* *p* < 0.05 considered statistically significant.

**Table 3:** Assessment of the caregiver practices regarding the administration of paracetamol and its association with demographic variables.

	Duration of Treatment (Days) N (%)			χ^2^ (df), *p*-Value	You Give Paracetamol Based on N (%)			χ^2^ (df), *p*-Value	Number of Times Paracetamol Is Given in 24 h. N (%)			χ^2^ (df), *p*-Value
	3	5	>5		Age	Body Weight	Severity of Fever		Every 3 h	Every 5 h	Every 6 h	
**Caregiver**				1.07 (4), *p* = 0.89				8.74 (4), *p* = 0.068				6.59 (4), *p* = 0.15
Mother	125 (33.5)	38 (10.2)	39 (10.5)		76 (20.4)	30 (8)	96 (25.7)		128 (34.3)	37 (9.9)	37 (9.9)	
Father	81 (21.7)	28 (7.5)	24 (6.4)		34 (9.1)	32 (8.6)	65 (18)		92 (24.7)	15 (4)	26 (7)	
Relatives/Nanny	20 (5.4)	10 (2.7)	8 (2.1)		16 (4.3)	7 (1.9)	15 (4)		19 (5.1)	9 (2.4)	10 (2.7)	
**Age (Years)**				7.82 (6), *p* = 0.25				1.25 (4), *p* = 0.86				4.13 (4), *p* = 0.38
18–30	50 (13.4)	17 (4.6)	15 (4)		28 (7.5)	17 (4.6)	37 (9.9)		54 (14.5)	17 (4.6)	11 (2.9)	
31–40	137 (36.7)	44 (11.8)	40 (10.7)		74 (19.8)	42 (11.3)	105 (28.2)		142 (38.1)	31 (8.3)	48 (12.9)	
Above 40	39 (10.5)	15 (4)	16 (4.3)		24 (6.4)	10 (2.7)	36 (9.7)		43 (11.5)	13 (3.5)	14 (3.8)	
**Education**				21.58 (4), *p* < 0.001 *				30.70 (4), *p* < 0.001 *				17.77 (4), *p* = 0.001 *
Up to primary education	16 (4.3)	13 (3.5)	15 (4)		5 (1.3)	6 (1.6)	33 (8.8)		30 (8)	7 (1.9)	7 (1.9)	
Intermediate	120 (32.2)	23 (6.2)	40 (10.7)		63 (16.9)	24 (6.4)	96 (25.7)		126 (33.8)	35 (9.4)	22 (5.9)	
Secondary and higher	90 (24.1)	40 (10.7)	16 (4.3)		58 (15.5)	39 (10.5)	49 (13.1)		83 (22.3)	19 (5.1)	44 (11.8)	

* *p* < 0.05 considered statistically significant.

**Table 4:** Assessment of caregiver practices in reviewing critical safety information on paracetamol labels.

	I Check the Directions for Use on the Label			χ^2^ (df)	*p*-Value	Warning on the Label		χ^2^ (df)	*p*-Value	AOR (95%CI)	*p*-Value
	Yes (%)	No (%)	Not Sure			Yes (%)	No (%)				
**Caregiver**				4.83	0.29			7.51 (2)	0.023 *		
Mother	166 (44.5)	12 (3.2)	24 (6.4)			115 (30.8)	87 (23.2)			1	
Father	99 (26.5)	13 (3.5)	21 (5.6)			57 (15.3)	76 (20.4)			1.21 (0.54–2.69)	0.64
Relatives Nanny/	28 (7.5)	2 (0.5)	8 (2.1)			16 (4.3)	22 (5.9)			1.88 (0.87–4.05)	0.10
**Age (Years)**				3.17 (4)	0.52			1.93 (2)	0.38		
18–30	61 (16.4)	7 (1.9)	14 (3.8)			36 (9.7)	46 (12.3)			0.73 (0.36–1.5)	0.40
31–40	180 (48.3)	15 (4)	26 (7)			114 (30.6)	107 (28.7)			0.99 (0.54–1.82)	0.98
Above 40	52 (13.9)	5 (1.3)	13 (3.5)			38 (10.2)	32 (8.6)			1	
**Education**				15.98 ^a^	0.002 *			61.60 (2)	0.001 *		
Up to primary education	28 (7.5)	10 (2.7)	6 (1.6)			11 (2.9)	33 (8.8)			0.11 (0.05–0.25)	0.001 *
Intermediate	144 (38.6)	8 (2.1)	31 (8.3)			67 (18)	116 (31.1)			0.19 (0.11–0.31)	0.001 *
Secondary and higher	121 (32.4)	9 (2.4)	16 (4.3)			110 (29.5)	36 (9.7)			1	

* *p* < 0.05 considered statistically significant. ^a^ Fisher exact test.

**Table 5:** Assessing the caregiver practices of checking the expiry date.

	Check Expiry Date		χ^2^ (df)	*p*-Value	AOR (95%CI)	*p*-Value
	Yes (%)	No (%)				
**Caregiver**			3.37 (2)	0.18		
Mother	155 (41.6)	47 (12.6)			1	
Father	94 (25.2)	39 (10.5)			0.65 (0.22–1.84)	0.41
Relatives/Nanny	32 (8.6)	6 (1.6)			0.71 (0.25–1.96)	0.51
**Age (Years)**			1.90 (2)	0.38		
18–30	58 (15.5)	24 (6.4)			0.69 (0.30–1.59)	0.39
31–40	172 (46.1)	49 (13.1)			1.0 (0.48–2.07)	0.98
Above 40	51 (13.7)	19 (5.1)			1	
**Education**			81.34 (2)	0.001 *		
Up to primary education	9 (2.4)	35 (9.4)			0.063 (0.027–0.14)	0.001 *
Intermediate	154 (41.3)	29 (7.8)			1.28 (0.71–2.29)	0.40
Secondary and higher	118 (31.6)	56 (15)			1	

* *p* < 0.05 considered statistically significant.

## Data Availability

This will be provided upon reasonable request.
